# *In vitro* enzymatic degradation of the PTMC/cross-linked PEGDA blends

**DOI:** 10.3389/fbioe.2023.1253221

**Published:** 2023-09-01

**Authors:** Wei Li, Meina Lin, Chenchao Wang, Yongping Lu, Yu Sui, Xiang Ni, Jing Guo, Miao Jiang, Liqun Yang, Hong Cui

**Affiliations:** ^1^ Liaoning Research Institute of Family Planning, The Affiliated Reproductive Hospital of China Medical University, Shenyang, China; ^2^ Department of Plastic Surgery, First Hospital of China Medical University, Shenyang, China; ^3^ Department of Obstetrics and Gynecology, Shengjing Hospital of China Medical University, Shenyang, China

**Keywords:** poly(trimethylene carbonate), PEGDA, UV cross-linking, enzymatic degradation, lipase

## Abstract

**Introduction:** Poly(1,3-trimethylene carbonate) (PTMC) is a flexible amorphous polymer with good degradability and biocompatibility. The degradation of PTMC is critical for its application as a degradable polymer, more convenient and easy-to-control cross-linking strategies for preparing PTMC are required.

**Methods:** The blends of poly(trimethylene carbonate) (PTMC) and cross-linked poly(ethylene glycol) diacrylate (PEGDA) were prepared by mixing photoactive PEGDA and PTMC and subsequently photopolymerizing the mixture with uv light. The physical properties and *in vitro* enzymatic degradation of the resultant PTMC/cross-linked PEGDA blends were investigated.

**Results:** The results showed that the gel fraction of PTMC/cross-linked PEGDA blends increased while the swelling degree decreased with the content of PEGDA dosage. The results of *in vitro* enzymatic degradation confirmed that the degradation of PTMC/cross-linked PEGDA blends in the lipase solution occurred under the surface erosion mechanism, and the introduction of the uv cross-linked PEGDA significantly improved the resistance to lipase erosion of PTMC; the higher the cross-linking degree, the lower the mass loss.

**Discussion:** The results indicated that the blends/cross-linking via PEGDA is a simple and effective strategy to tailor the degradation rate of PTMC.

## 1 Introduction

Poly(1,3-trimethylene carbonate) (PTMC) is a flexible amorphous polymer with good degradability and biocompatibility (Hou et al., 2021; Hou et al., 2022) while without acidic degradation substances, thus avoiding severe inflammatory reactions (Yang et al., 2014a; Yang et al., 2015). Therefore, PTMC has a wide range of applications in biomedicine, such as drug delivery systems (Fukushima, 2016; Mohajeri et al., 2020; Hou et al., 2023) and tissue engineering (Li et al., 2020; Brossier et al., 2021; He et al., 2021).

The degradation of PTMC is critical for its application as a degradable polymer. Studies have shown that the high molecular weight PTMC has good morphological stability, but the degradation is too fast. On the contrary, low molecular weight PTMC degrades slowly (Yang et al., 2015). However, it has poor morphological stability and creeps quickly at room temperature (Yang et al., 2015). Therefore, suitable methods for modifying PTMC must be found to obtain polymers with controllable degradation time and stable morphologies. Numerous studies have shown that copolymerization can modulate the physical properties and control the degradation rate of PTMC. As Yang (Yang et al., 2014a) and Hou (Hou et al., 2019) reported, the appropriate introduction of hydrophobic or semi-crystalline segments into the structure reduces the degradation rate of PTMC. Although it is an effective strategy to adjust the degradation rate of PTMC, the degradation rate of copolymers is also affected by several factors, such as the molar ratio (Hou et al., 2022), molecular weight (Hou et al., 2020) of copolymers, etc., resulting in the controlled regulation of the degradation rate of PTMC still faces challenges.

The construction of biodegradable cross-linked networks (BCNs) is one of the efficient ways to retard polymer degradation and maintain morphological stability. Chemical cross-linking is a promising strategy to prevent the degradation of PTMC, and for example, Yang et al. prepared bis-TMC with a structure similar to TMC as a chemical cross-linker to obtain PTMC-BCNs (Yang et al., 2013; Yang et al., 2014b). The resulting PTMC-BCNs showed improved morphological stability and lower degradation rate *in vitro* (Hou et al., 2017) and *in vivo* (Yang et al., 2016). However, the difficulty of forming BCNs after chemical cross-linking severely limits their wide application.

Compared with chemical cross-linking, irradiation cross-linking allows PTMC to be molded before cross-linking. In particular, gamma- and electron beam irradiation enables rapid cross-linking of the molded PTMC (Liu et al., 2021a; Liu et al., 2021b; Jozwiakowska et al., 2011), and the cross-linked PTMC also has a slow degradation rate due to the formation of the cross-linked network (Bat et al., 2009; Bat et al., 2010a; Liu et al., 2021c), which can significantly improve the erosion resistance of PTMC. Grijpma et al. reported that biodegradable elastomeric PTMC-BCNs could be efficiently formed by gamma irradiation of the linear polymer in the presence of pentaerythritol triacrylate (PETA), and the enzymatic erosion rates of the networks could be decreased from 12.0 ± 2.9 to 3.0 ± 1.6 µm/day (Bat et al., 2010b). However, the way of irradiation cross-linking also has specific problems, for example, chain scission that simultaneously occurs with cross-linking, which can make the molecular weight of PTMC decrease (Yang et al., 2014b; Liu et al., 2021b), and the resulting low-molecular weight segments will form defects in the three-dimensional network, and reduce the performance of PTMC-BCNs.

UV photo-cross-linking is a promising solution to the problems of the above strategies. Flexible, elastomeric, and biodegradable networks could be readily prepared by UV irradiating PTMC films (Rongen et al., 2016; Zant and Grijpma, 2016; Wang et al., 2020). The design and preparation of prepolymers functionalized with double bonds are essential for UV photo-crosslinking of PTMC, such as the molecular weight of the prepolymer, the copolymerization ratio, and the number of double bonds can affect the degradation rate of the cross-linked PTMC. This will undoubtedly increase the factors affecting the degradation rate of UV-crosslinked PTMCs. Hence, more convenient and easy-to-control cross-linking strategies for preparing PTMC-BCNs are required.

Currently, poly (ethylene glycol) diacrylate (PEGDA) has been widely used as cross-linking agent (Kedzierska et al., 2022). PEGDA is a derivative of polyethylene glycol (PEG) with double-bonded acrylate groups at both ends, which can form various cross-linked networks through photo-polymerization in the presence of a photoinitiator. PEGDA is a hydrophilic material with low cytotoxicity and good biocompatibility (Warr et al., 2020). Therefore, multiple combinations of PEGDA-based materials have been developed for biomedical applications (Qin et al., 2021; Soriente et al., 2021).

In this study, PTMC/cross-linked PEGDA blends were prepared directly by UV cross-linking strategy using PEGDA as a cross-linking agent without preparing prepolymers. The thermal properties of the resulting PTMC/cross-linked PEGDA blend with different degrees of cross-linking were investigated. The *in vitro* enzymatic degradation behavior of PTMC/cross-linked PEGDA blends was also performed in lipase solutions to investigate the effect of cross-linked PEGDA on the degradation rate of PTMC.

## 2 Materials and methods

### 2.1 Materials

Trimethylene carbonate (TMC) was purchased from Daigang Biomaterial Co., Ltd (Jinan, Shandong, China), recrystallized twice with ethyl acetate, and dried to constant weight before polymerization. Stannous octoate [Sn(Oct)_2_] (95%) and lipase from Aspergillus oryzae (≥10 0,0 0 0 U/g) were obtained from Sigma-Aldrich and used as received. PEGDA (average Mw = 400Da) and 2-hydroxy-4-(2-hydroxyethoxy)-2-methylpropiophenone (I2959) were purchased from Innochem (Beijing, China). All other solvents and reagents were analytical grade and used without further purification.

### 2.2 Measurements

A Thermo Scientific Nicolet iS50 Fourier transforms infrared spectrometer (Madison, WI, USA) with an ATR accessory was used to analyze the chemical structure of the samples. The film sample was placed on the ATR accessory’s crystal face, and the samples’ infrared spectrum was collected. The testing range was 400–4,000 cm^−1^, the number of scans was 32, and the resolution was 4cm^−1^. The hydrophilicity of the PTMC/cross-linked PEGDA blend films was tested using a DSA25 drop-shape analyzer (Kruss, Hamburg, Germany). The test temperature was room temperature, and the test time was 3 s. A camera recorded the shape of ultrapure water droplets (5 μL) on the films, and the data were read by the computer. At least three positions were measured for each sample, and the average was taken. The glass transition temperature (Tg) of the PTMC/cross-linked PEGDA blends was determined using a Netzsch DSC 200 F3 (Netzsch, Selb, Germany) equipped with a liquid nitrogen cooling system. The measured temperature range was from −50°C to 100°C, and the heating rate was 10°C/min under a nitrogen atmosphere. The thermal stability of the PTMC/cross-linked PEGDA blends was performed using a Netzsch TGA 209 F3 (Netzsch, Selb, Germany) at a heating rate of 10°C/min from room temperature to 700°C under a nitrogen atmosphere. The temperature was set at Td when the mass loss was 5%. Before and after degradation, the films were photographed with a camera to obtain the macroscopic morphology of the material. The microscopic morphology of the films was obtained using a TESCAN MIRA LMS scanning electron microscope (SEM) (Brno, South Moravia, Czech Republic). Gold was sprayed on the surface of the materials to increase their electrical conductivity before testing.

### 2.3 PTMC synthesis

PTMC was synthesized by bulk ring-opening polymerization of TMC using Sn(Oct)_2_ as a catalyst (Li et al., 2020). In brief, dried TMC (30 g, 0.29 mol) and an anhydrous toluene solution of Sn(Oct)_2_ (0.2 M; 1/20000 eq, 2.9 μL) were added to the ampoule. After purging with dry nitrogen, the ampoule was heat-sealed under a vacuum (5 mmHg) and put in an oil bath at 130°C ± 2°C for 24 h. After the reaction, the ampoule was cooled to room temperature and smashed to obtain the crude polymer, which was dissolved in dichloromethane, followed by purification in ice methanol. The purified PTMC was vacuum dried to constant weight.

### 2.4 Preparation of PTMC/cross-linked PEGDA blends

**FIGURE 1:**
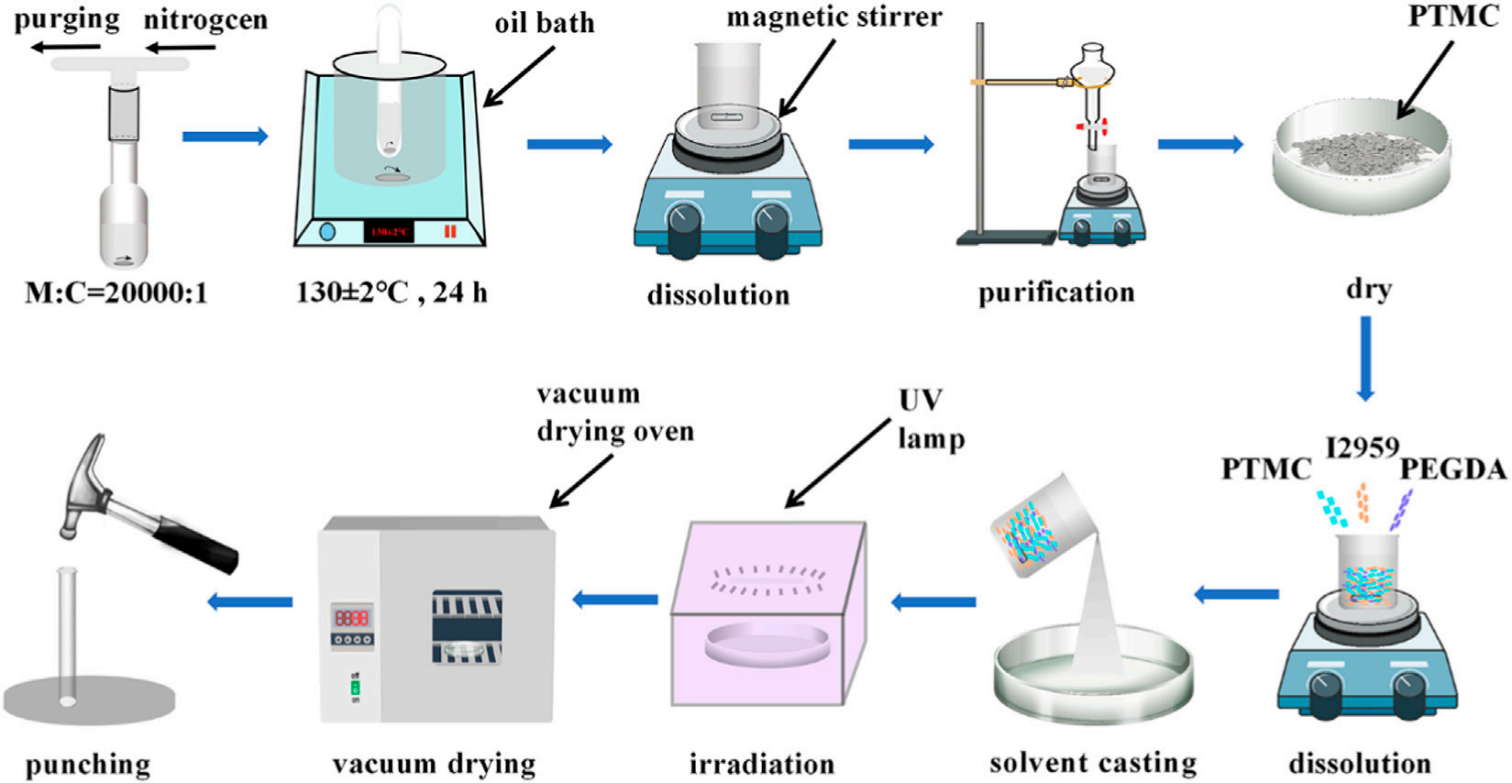
Schematic representation of the preparation of PTMC/cross-linked PEGDA blends.

Briefly, PTMC, PEGDA, and I2959 (photoinitiator) are dissolved in appropriate dichloromethane according to a predetermined weight ratio and poured into a glass plate (diameter: 9 cm, height: 1.5 cm). The glass plate was placed in a fume hood to allow the solvent to evaporate naturally. After evaporation, the films were irradiated under a UV lamp (365 nm) for 3 min. The photo-crosslinked blends were vacuum dried to constant weight, and then fixed-size circular films were punched (diameter: 7 mm) for subsequent experiments. The preparation process of PTMC/cross-linked PEGDA blends is shown in Figure 1.

### 2.5 Gel fraction and swelling degree

Samples were weighed and stored in a glass vial containing dichloromethane for 5 days to determine the gel fraction and swelling degree (SR) of PTMC/cross-linked PEGDA blends. The solvent was changed once a day to remove the soluble fraction altogether. After 5 days, the swollen gel was taken out, and the wet weight of the sample was weighed. Then, the piece was dried to a constant weight using a vacuum-drying oven. The gel fraction and swelling degree were calculated using Eqs 1, 2.

$$Gel\;fraction\;\left(\%\right)=\frac{m}{M}\times 100$$ (1)

$$SR\;\left(\%\right)=\frac{M_{t}-M}{M}\times 100$$ (2)

M denotes the initial weight of PTMC/cross-linked PEGDA blends before swelling, Mt is the wet weight of PTMC/cross-linked PEGDA blends after 5 days of swelling, and m represents the dry weight of PTMC/cross-linked PEGDA blends after swelling. Three replicate samples were set to measure gel fraction and swelling ratio, and the results were averaged.

### 2.6 *In vitro* enzymatic degradation

Before degradation, the initial weight (Wi) of the circular films of PTMC/cross-linked PEGDA blends were weighed using an electronic balance (Sartorius, German). The initial thickness (Ti) of the film was recorded by electronic digital calipers (Guanglu, China). Then, the circular films were placed in a glass tube containing 500 μL lipase solution that was changed every 4 days. The glass tubes were placed in a constant temperature oscillator at 37°C and gently shaken daily for 8 h. Every 4 days, three parallel samples were taken from the lipase solutions in each group and washed with deionized water. Water from the surface of the samples was absorbed with absorbent paper, and the films were weighed and thickness measured, then dried under vacuum at 37°C to a constant weight for further analysis. Before and after degradation, the pH of the lipase solutions was monitored using a Toledo-Mettler InLabMicro TM pH meter equipped with a three-in-one microelectrode (Toledo-Mettler, Zurich, Switzerland). The mass loss, thickness loss, and water uptake of the samples were calculated according to Eqs 3–5, respectively. The degradation rate constant k was used to quantify the mass loss for each sample set and calculated according to Eq. 6 (Yang et al., 2015; Hou et al., 2021).

$$Mass\;loss\;\left(\%\right)=\frac{W_{i}-W_{d}}{W_{i}}\times 100$$ (3)

$$Loss\;in\;thickness\;\left(\%\right)=\frac{T_{i}-T_{d}}{T_{i}}\times 100$$ (4)

$$water\;uptake\;\left(\%\right)=\frac{W_{w}-W_{d}}{W_{d}}\times 100$$ (5)

$$M_{t_{2}}=M_{t_{1}}-k\left(t_{2}-t_{1}\right)$$ (6)

Wi is the initial weight of the sample before degradation, Ww is the wet weight of the sample, and Wd is the dry weight after degradation. Ti is the initial thickness of the sample before degradation, and Td is the thickness of the dried film after degradation. Mt is the mass loss at degradation time t.

## 3 Results and discussion

### 3.1 Preparation of PTMC/cross-linked PEGDA blends

PTMC/cross-linked PEGDA blends with different degrees of cross-linking were prepared by increasing the amount of PEGDA with a given content of PTMC and I2959, as shown in Table 1.

**TABLE 1:** PTMC/cross-linked PEGDA blends with different PEGDA contents.

	PTMC(g)	PEGDA(g)	I2959(g)
N_1_	1.5	0	0
N_2_	1.5	0.3	0.0075
N_3_	1.5	0.6	0.0075
N_4_	1.5	0.9	0.0075
N_5_	0	0.6	0.0075

**FIGURE 2:**
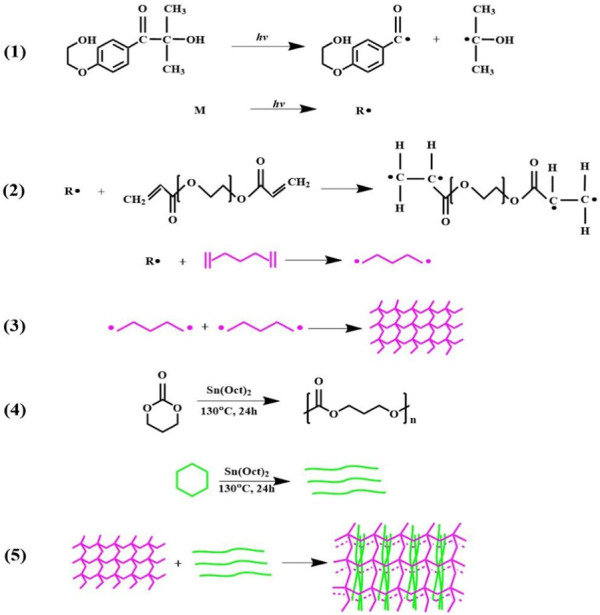
Schematic representation of PTMC/cross-linked PEGDA blends formation. 1) I2959 generates free radicals under UV light irradiation; 2) reaction between PEGDA and free radicals to form active centers; 3) activated PEGDA bonds together to form a self-crosslinking network; 4) ring-opening polymerization of TMC to form linear PTMC; 5) linear PTMC linear PTMC chains intertwine or intersperse with the cross-linked PEGDA, forming a three-dimensional network of cross-linked PTMC.

During the cross-linking process, I2959 generates free radicals in the presence of UV light. The radicals attack the -C=C- of PEGDA, creating a reactive center and initiating the self-crosslinking of PEGDA to form a three-dimensional cross-linked structure. The cross-linked PEGDA acts as a physical cross-linking point for the linear PTMC, prompting the linear PTMC chains to intertwine or intersperse with the cross-linked PEGDA, thus forming a three-dimensional network of cross-linked PTMC. The formation of PTMC/cross-linked PEGDA blends is shown in Figure 2.

### 3.2 Structural characterization

**FIGURE 3:**
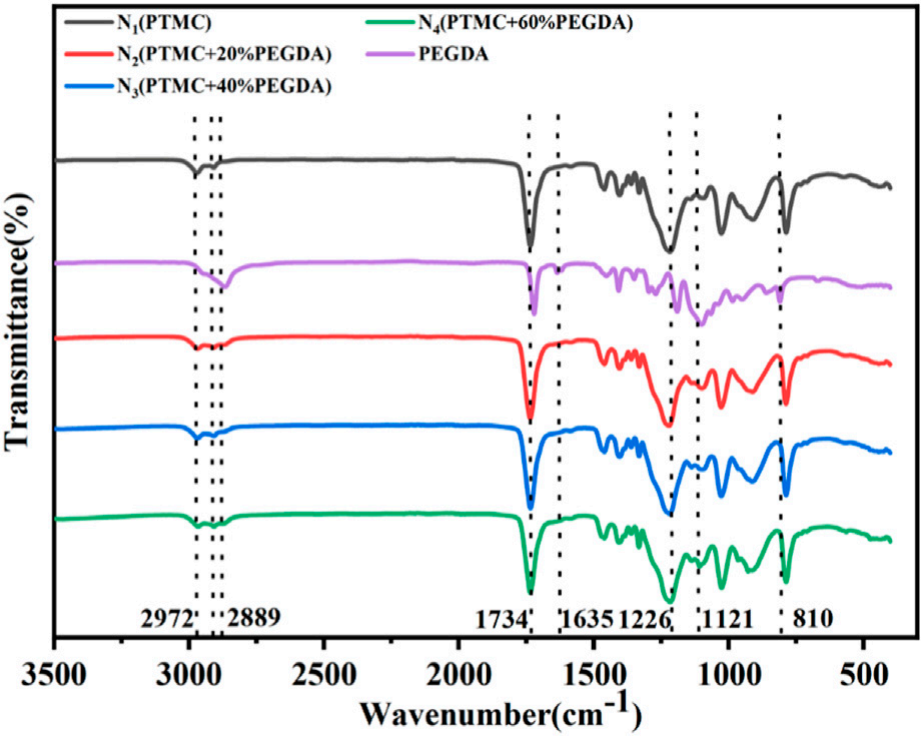
FTIR spectra of PTMC, PEGDA monomer, and PTMC/cross-linked PEGDA blends.

As shown in Figure 3, the FTIR spectra of the pure PTMC, pure PEGDA, and PTMC/cross-linked PEGDA blends were compared to identify the chemical alteration. In PEGDA, the peak at 1,121 cm^−1^ is the symmetric stretching vibration of C-O-C, and the peak at 2,889 cm^−1^ is the symmetric stretching vibration absorption peaks of C-H in -CH_2_-CH_2_- segments. As for the spectrum of PEGDA, it should be noted that the peaks at 1,635 and 810 cm^−1^ confirmed the presence of alkene moieties. The disappearance of these peaks indicates the curing of acrylates, and PEGDA is successfully self-crosslinked under UV light. The C=O stretching vibration absorption peaks of PEGDA and PTMC is at 1720 cm^−1^ and 1734 cm^−1^, respectively, which overlaps in the PTMC/cross-linked PEGDA blends. In PTMC, the absorption peaks caused by the asymmetric and symmetric stretching vibrations of -CH_2_- at 2,972 cm^−1^ and 2,910 cm^−1^ can also be detected. Compared with the PTMC and PEGDA spectra, the PTMC/cross-linked PEGDA blends did not show any apparent new absorption peak, indicating no chemical reaction between the two polymers.

### 3.3 Gel fraction and swelling degree

**FIGURE 4:**
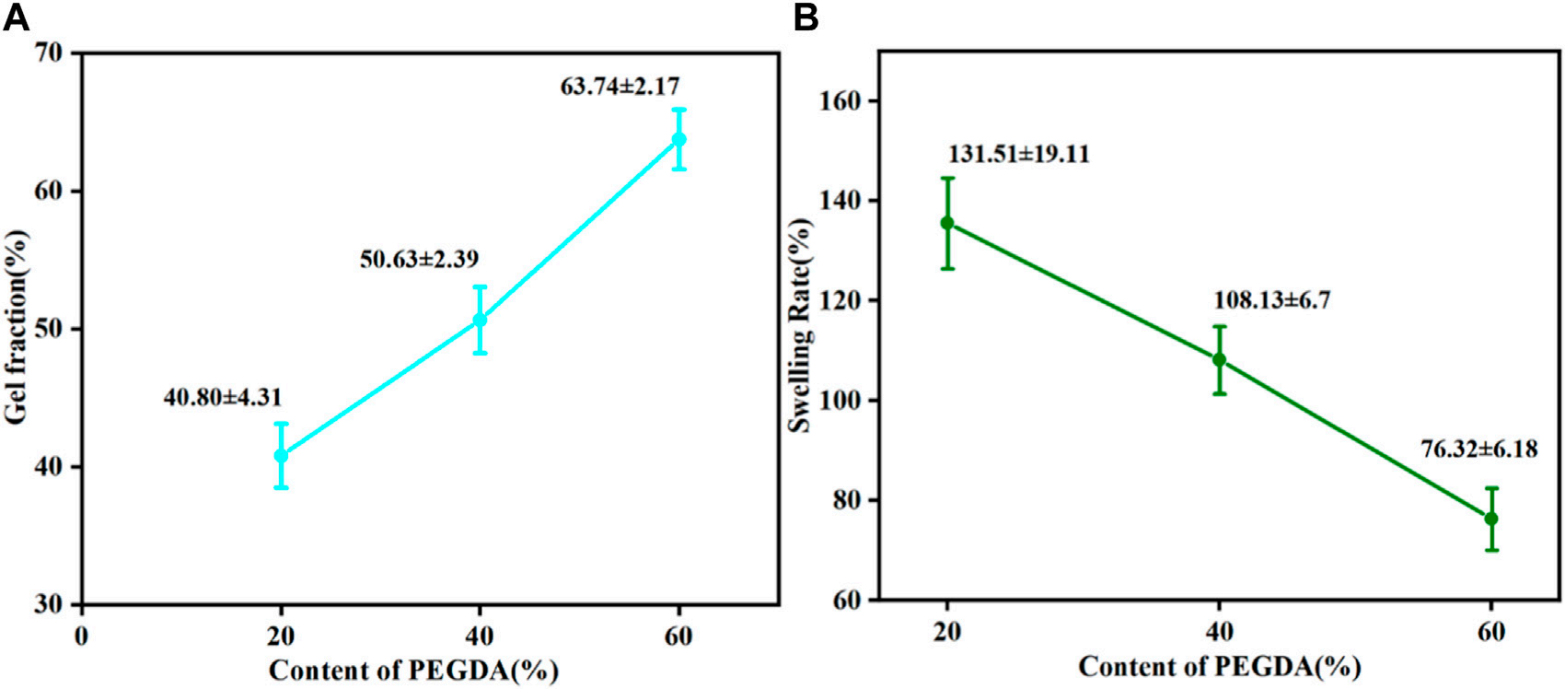
Gel fraction **(A)** and swelling degree **(B)** of PTMC/cross-linked PEGDA blends.

The gel fraction can be used to indicate the degree of cross-linking. Obviously, the gel fraction gradually became more prominent while the swelling degree decreased with the increase of PEGDA, as shown in Figure 4, indicating that the degree of cross-linking of the PTMC/cross-linked PEGDA blends gradually increased. This result suggests that the increase in PEGDA content can lead to the formation of a denser network.

### 3.4 Thermal properties

**FIGURE 5:**
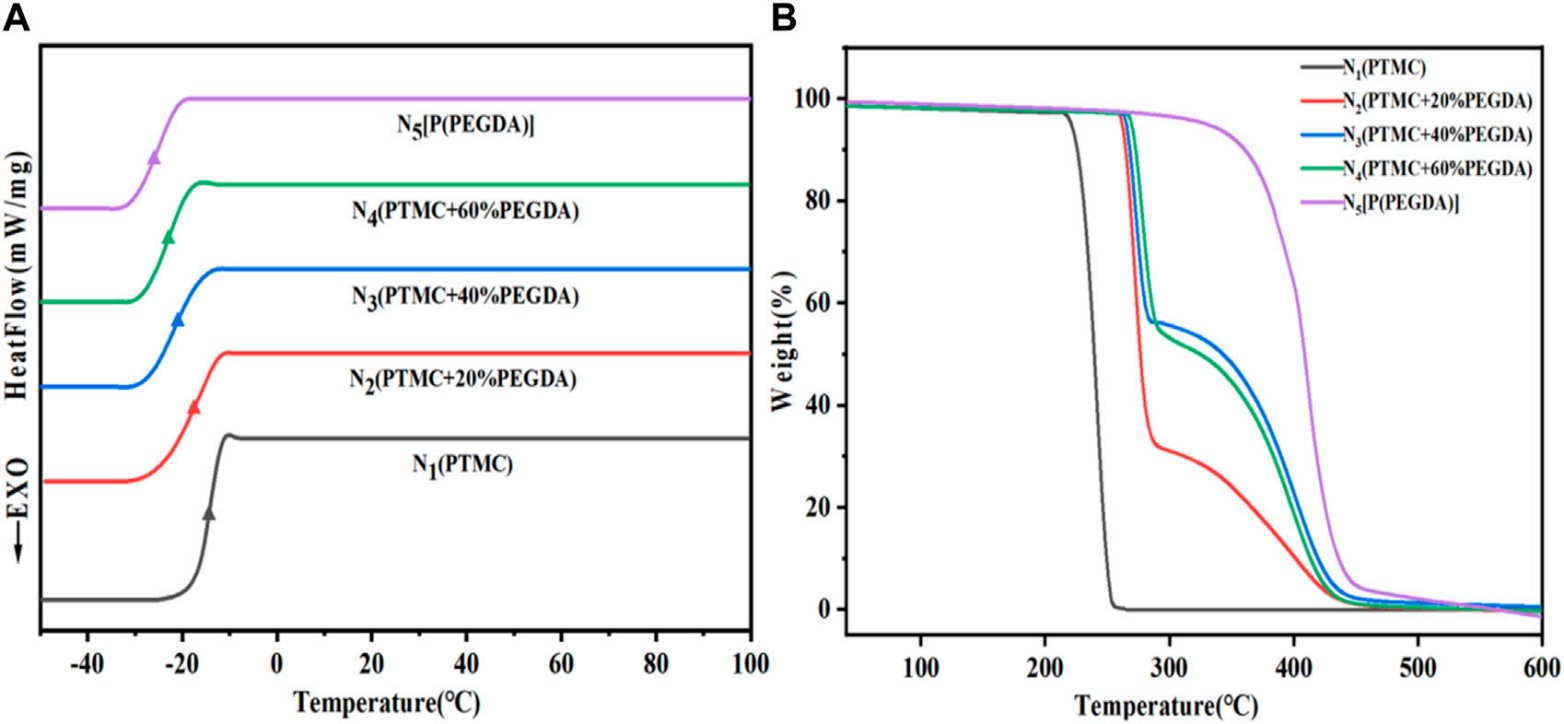
DSC **(A)** and TGA **(B)** curves of obtained PTMC/cross-linked PEGDA blends.

As shown in Figure 5A and Table 2, the Tg of PTMC is -16.6°C and the Tg of UV cross-linked P(PEGDA) is –27.2°C. With increased PEGDA content in the feeding ratio, the Tg of PTMC/cross-linked PEGDA blends showed a decreasing trend. It is attributed to the plasticization effect of P(PEGDA) in the blends (Fang et al., 2014; Yang et al., 2019). With the increase of the number of flexible [-O-CH_2_-CH_2_ -] chains from P(PEGDA) in PTMC/cross-linked PEGDA blends, the motion of molecular chains is relatively active, resulting in lower Tg.

**TABLE 2:** Thermal properties of PTMC/cross-linked PEGDA blends.

	N_1_	N_2_	N_3_	N_4_	N_5_
Tg (°C)	-16.6	-19.9	-21.6	-23.6	-27.2
Td (°C)	239.5	263.6	264.4	265.4	329.9

As shown in Figure 5B and Table 2, The thermal decomposition temperature of PTMC is about 240°C. The thermal decomposition temperature of P(PEGDA) is higher than that of PTMC, about 330°C. With the increase of PEGDA, the thermal stability of PTMC/cross-linked PEGDA blends gradually increases due to the formation of a cross-linked network.

### 3.5 Hydrophilicity of PTMC/cross-linked PEGDA blends

**FIGURE 6:**
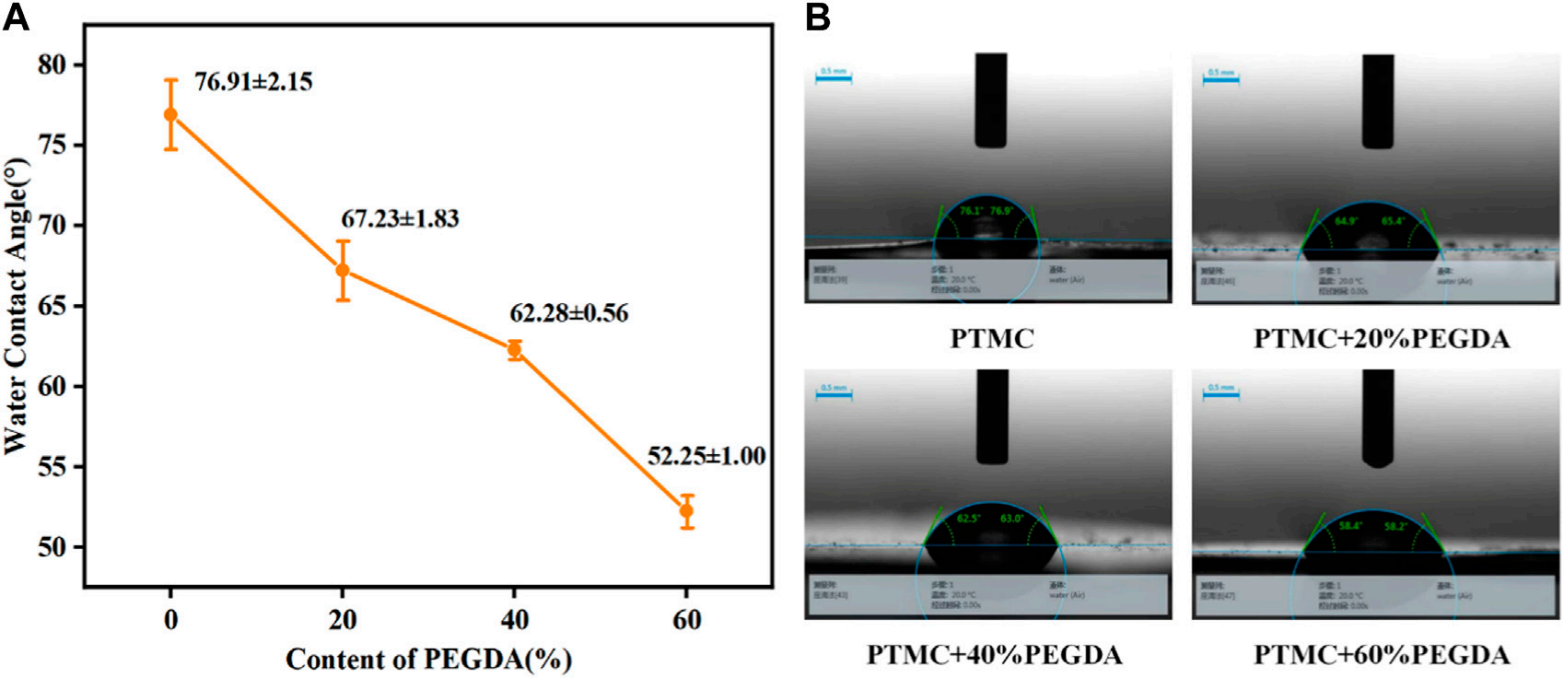
The water contact angle of PTMC/cross-linked PEGDA blends.

Hydrophilicity is an essential factor in determining material properties. The water contact angle is an important parameter to measure the wettability of liquid to materials. A static water contact angle measurement was used to investigate the hydrophilicity of PTMC/cross-linked PEGDA blends, as shown in Figure 6. It can be seen from Figure 6A that the water contact angle of PTMC is 76.91° ± 2.15°, which shows the hydrophobic nature. With the increase of PEGDA dosage, the water contact angle gradually becomes smaller, indicating the hydrophilicity of the material is improved. It suggests that the introduction of hydrophilicity of PEGDA improves the PTMC blends.

### 3.6 *In vitro* enzymatic degradation of PTMC/cross-linked PEGDA blends films

#### 3.6.1 Mass loss and thickness loss

**FIGURE 7:**
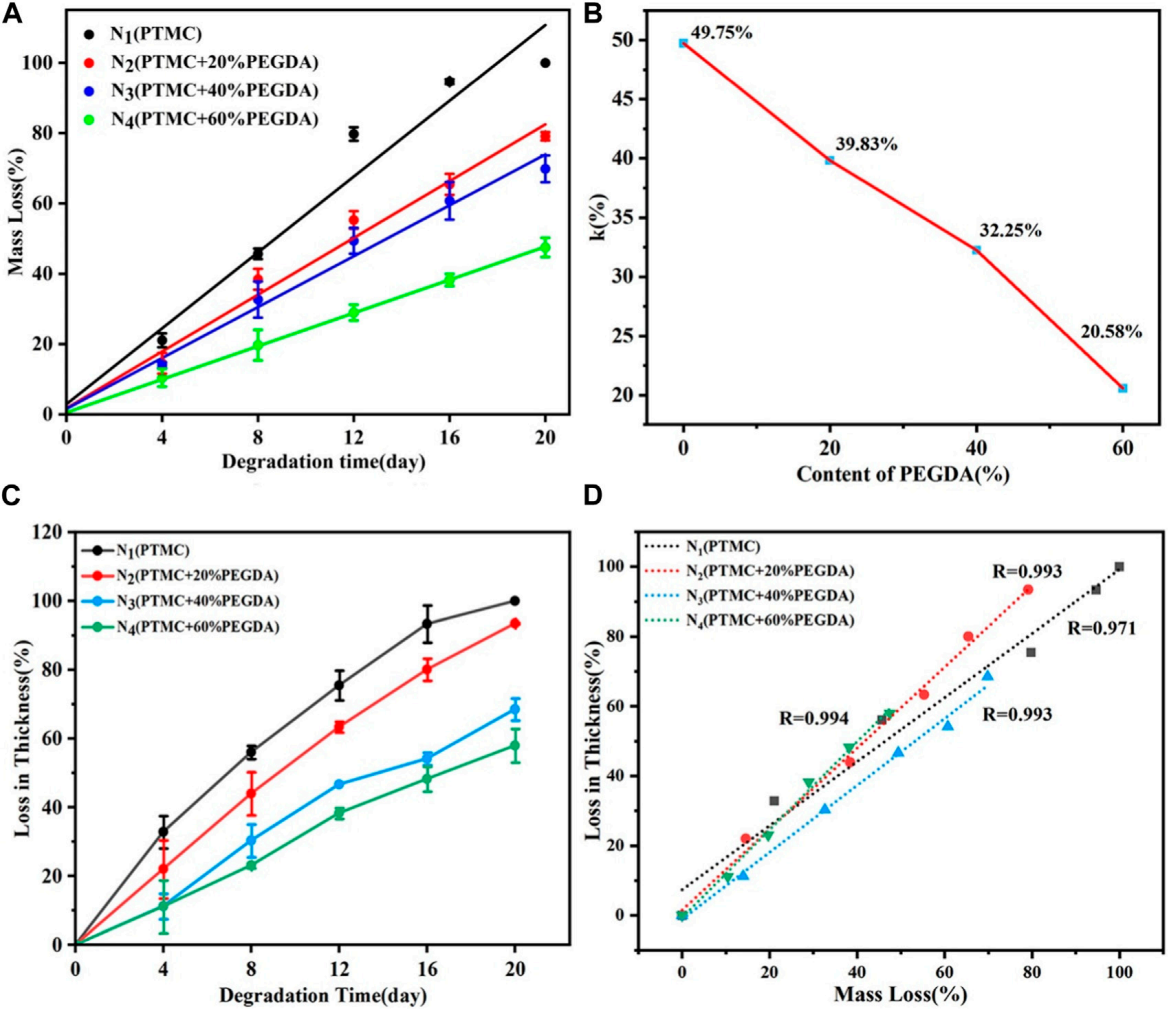
Mass loss **(A)**, degradation rate constant k **(B)** and thickness loss **(C)** of PTMC/cross-linked PEGDA as a function of degradation time, and thickness loss as function of mass loss **(D)** of PTMC/cross-linked PEGDA.

Mass loss is one of the most critical parameters in studying the degradation properties of biodegradable polymers. The degradation behavior of PTMC/cross-linked PEGDA blends is shown in Figure 7. Figure 7 (a) shows an excellent linear relationship between mass loss and degradation time for each group. The mass loss of PTMC was nearly 100% after 20 days, while the mass loss of the N_4_ group (PTMC +60% PEGDA) was only 30% of the initial weight. The results showed that the degradation rate of PTMC/cross-linked PEGDA blends decreased with the increase of PEGDA, which significantly prolonged the degradation period of the materials. As shown in Figure 7B, the degradation rate constant k of PTMC is 49.75%. With the increase of PEGDA, the k value showed a downward trend, indicating that the degradation period of the films became longer. Blending with UV cross-linked PEGDA can effectively slow down the degradation of PTMC. We also investigated the thickness loss of the films as a function of the degradation time, as shown in Figure 7C. The thickness of the films gradually decreases with time. It can be seen from Figure 7D that the mass loss of the film is linearly related to the thickness loss, and both occur simultaneously. This confirms that the degradation of PTMC/cross-linked PEGDA occurred via surface erosion mechanisms.

#### 3.6.2 Macroscopic and microscopic morphology

**FIGURE 8:**
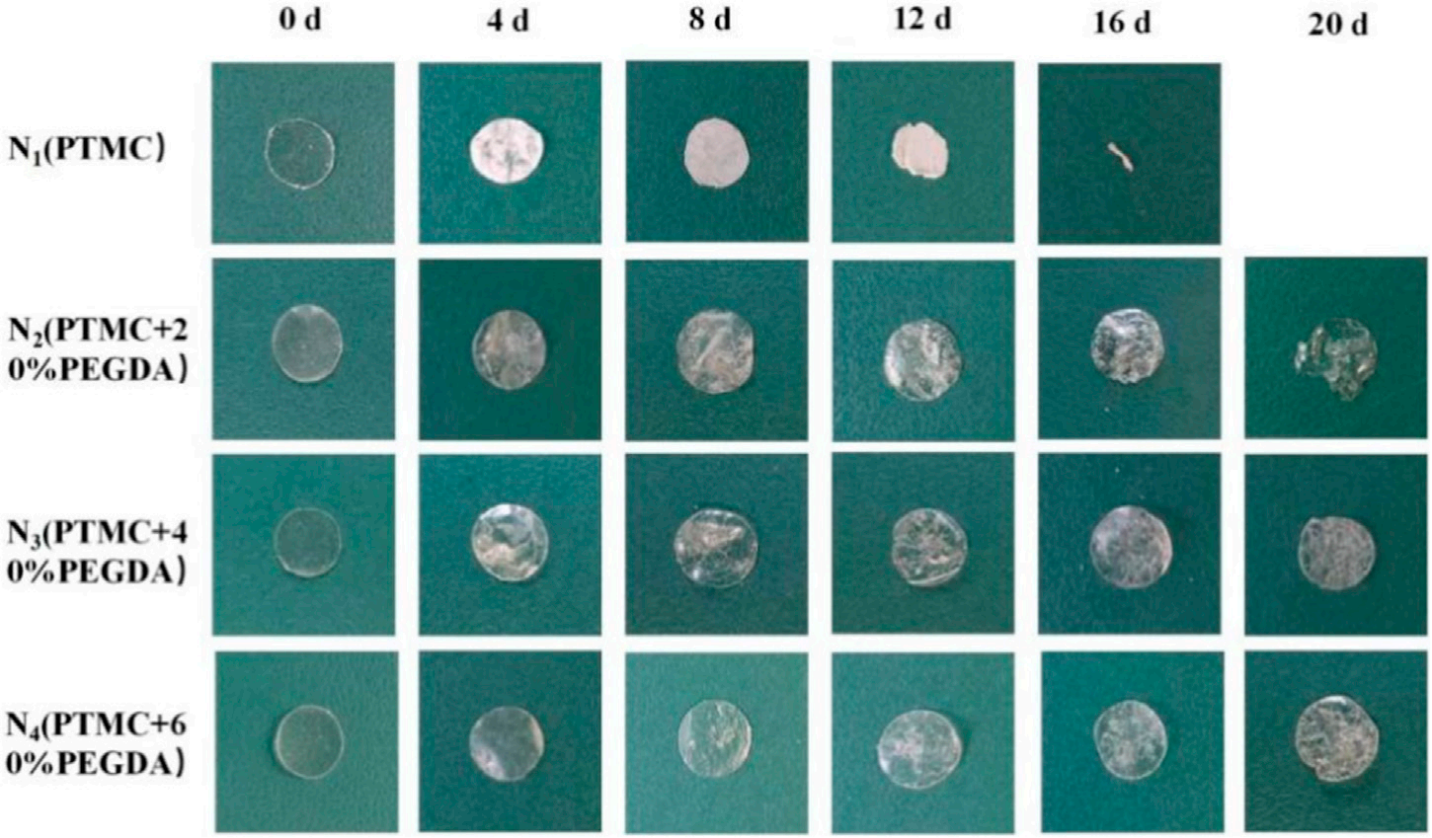
The macroscopic morphology of films before and after enzymatic degradation.

Photographing samples at different degradation times obtained the macroscopic morphology of the material to determine the stability of the PTMC/cross-linked PEGDA blends. As shown in Figure 8, for the N_1_(PTMC) group, the original morphology could not be maintained after degradation of 16 days, and it was degraded entirely at day 20. The N_2_ group samples remained in their original shape on day 16. Films were still present on day 20, but the integrity of the films was lost. On day 20, the films of groups N_3_ and N_4_ retained their integrity and original morphology without curls. However, the films of both groups developed cracks during degradation, which appeared earlier and more severe in the N_3_ group than in the N_4_ group. It can be seen that the network structure formed after cross-linking increases the stability of the films, reduces the deformation of the materials, and slows down the degradation.

**FIGURE 9:**
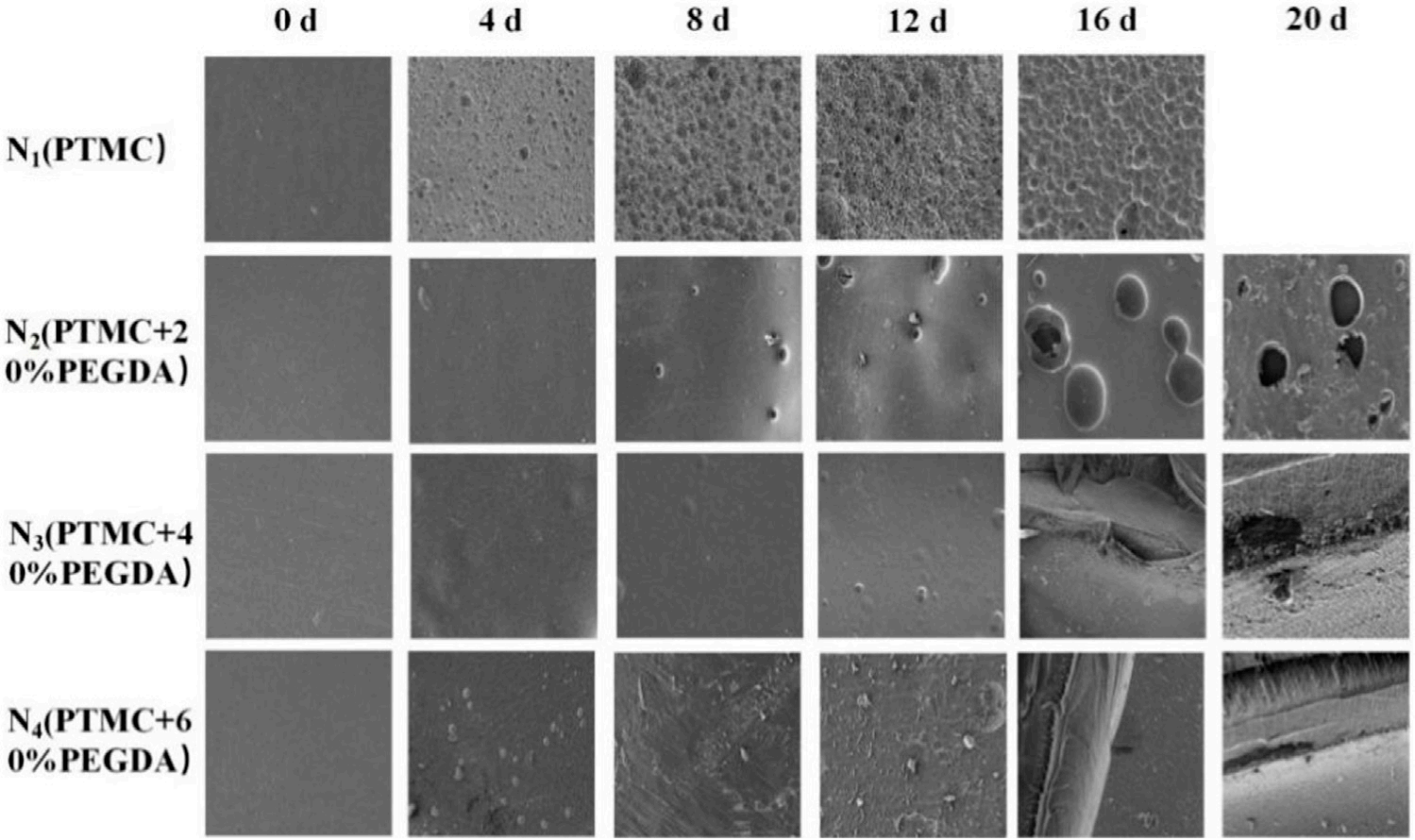
The surface morphology of films before and after enzymatic degradation.

Figure 9 shows the microscopic surface morphology of the films during degradation. Before degradation, the surfaces of the films are all flat and smooth. A large number of holes appear on the surface of PTMC after degradation. As the degradation time increases, the number of pores and holes and their size gradually increases. Holes also appeared on the surface of the N_2_ group films, but the number and size of the holes were smaller than those of PTMC. Fewer holes appear in the N_3_ group, and many cracks appear in the later stages of degradation. In the early stage of degradation, no holes appeared in the N_4_ group. As the degradation progressed, cracks also appeared in the films of the N_4_ group, but the distribution and size were not as large as those of the N_3_ group. The results indicate that the formation of cross-linked networks effectively prevents degradation.

#### 3.6.3 Water uptake of films

**FIGURE 10:**
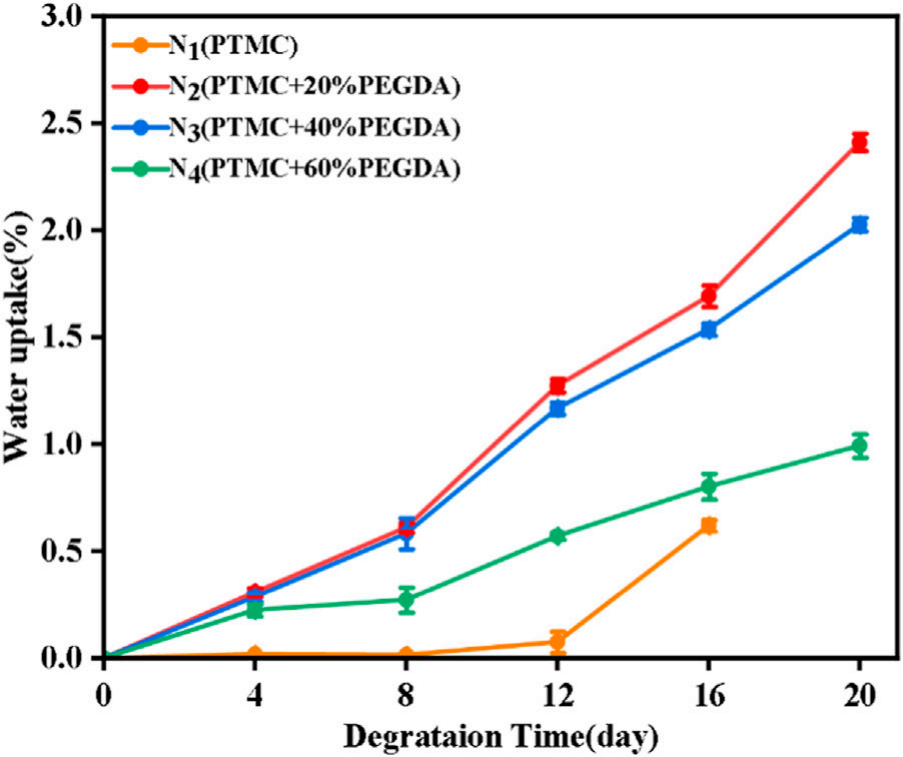
The water uptake of films during enzymatic degradation.

As shown in Figure 10, the water uptake of the PTMC/cross-linked PEGDA blends gradually increased with the degradation time. As a surfactant, lipase disperses the degradation products into solution, thus accelerating the degradation of the PTMC/cross-linked PEGDA blend (Yang et al., 2015), leading to pores and holes on the material’s surface. In the later stages of degradation, the pores and holes became larger. The appearance of pores promotes the storage of water. Although PTMC degrades the fastest and has many pores on its surface, the PEGDA chain segments significantly increase the hydrophilicity of the PTMC/cross-linked PEGDA mixture. The higher the PEGDA content, the more hydrophilic the PTMC/cross-linked PEGDA mixture becomes, which gradually increases its water absorption during degradation.

#### 3.6.4 pH value

**FIGURE 11:**
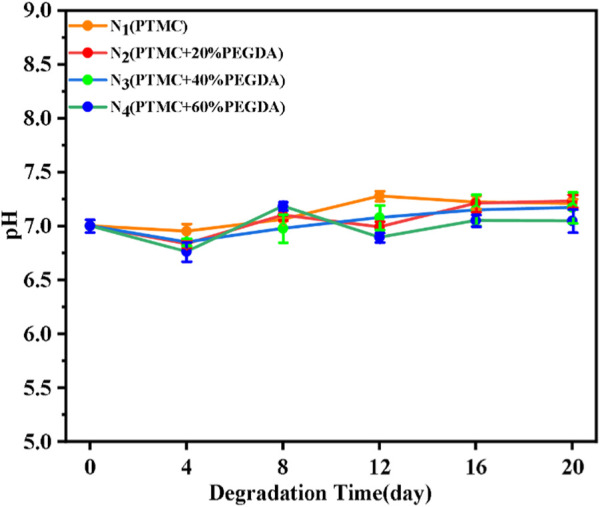
The changing trend in the pH value of films during enzymatic degradation.

During the degradation process, we monitored the lipase solution’s pH variation as a function of the degradation time. It has been shown that PTMC does not produce acidic degradation products during enzymatic degradation and is independent of its relative molecular weight (Yang et al., 2015). As shown in Figure 11, the pH of the PTMC/cross-linked PEGDA blends remained stable during the degradation process. This result indicates that the degradation of the PTMC/cross-linked PEGDA blends did not produce acidic degradation products, thus avoiding the development of local inflammation and having a more significant potential for biomedical applications.

#### 3.6.5 The change in thermal properties

**FIGURE 12:**
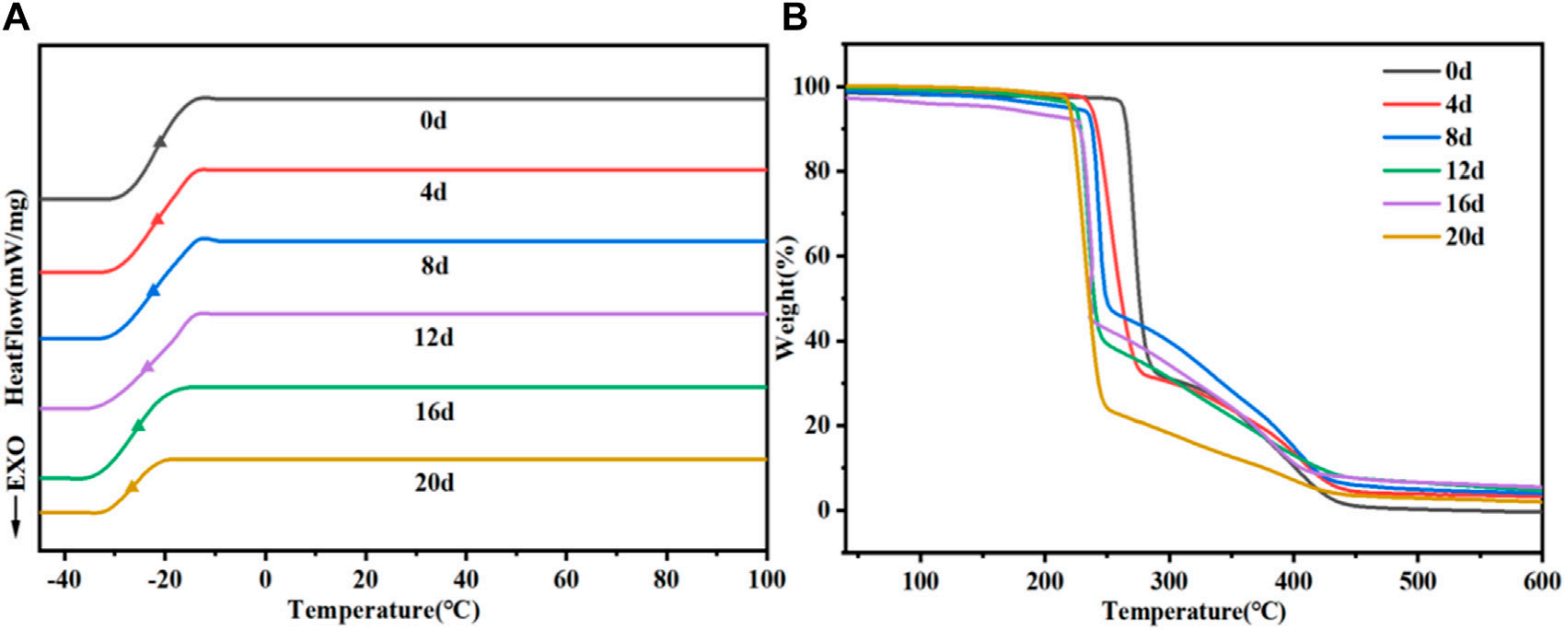
Changes in DSC **(A)** and TGA **(B)** of PTMC/cross-linked PEGDA blends N_3_ during degradation.

We also examined the changes in the thermal properties of PTMC/cross-linked PEGDA blends during degradation and illustrated with the N_3_ group as a typical representative, as shown in Figure 12 and Table 3. As shown in Table 3, the Tg tends to decrease as the degradation proceeds. The breakage of molecular chains leads to a higher degree of motion, and Tg decreases accordingly. The destruction of the cross-linked structure due to degradation causes the polymer to lose thermal stability, which leads to a decrease in the Td. Furthermore, the accumulation of degradation products on the material’s surface also decreases its thermal stability and enhances a lower Tg due to the plastic effect of the degradation products.

**TABLE 3:** The change of Tg and Td during degradation.

	0d	4d	8d	12d	16d	20d
Tg (°C)	-21.6	-21.8	-23.1	-24.9	-26.5	-27.6
Td (°C)	263.6	237.3	224.6	220.5	219.2	163

## 4 Conclusion

In this study, PTMC/crosslinked PEGDA blends were prepared directly using UV crosslinking strategy with PEGDA as crosslinking agent. The degradation behavior of the PTMC/cross-linked PEGDA blends was characterized to assess their potential as biomaterials. The gel fraction of the PTMC/cross-linked PEGDA blends became more prominent with increasing PEGDA. The degradation mechanism of the PTMC/cross-linked PEGDA blends was surface erosion. The degradation rate of the PTMC/cross-linked PEGDA blends decreased significantly with increasing cross-linkage. The pH of the enzyme solution confirmed that the PTMC/cross-linked PEGDA blend degraded without acid production. Hence, the introduction of cross-linked PEGDA to fabricate the PTMC/cross-linked PEGDA blend is a simple and effective strategy to tailor the degradation rate of PTMC. This strategy eliminates the preparation of prepolymers, which reduces the influencing factors affecting the degradation of the blends and avoids defects such as scission of molecular chain due to irradiation. More importantly, this strategy can meet the practical needs of more convenient and easy-to-control cross-linking for PTMC, which can help to enrich the in-depth study of cross-linked PTMC.

## Data Availability

The original contributions presented in the study are included in the article/Supplementary Material, further inquiries can be directed to the corresponding authors.
